# The Impact of a Mnemonic Acronym on Learning and Performing a Procedural Task and Its Resilience Toward Interruptions

**DOI:** 10.3389/fpsyg.2019.02522

**Published:** 2019-11-06

**Authors:** Tara Radović, Dietrich Manzey

**Affiliations:** Institute of Psychology and Ergonomics, Berlin Institute of Technology, Berlin, Germany

**Keywords:** interruptions, sequential task, resumption time, goal activation, sequential error, mnemonic technique, acronym, procedure learning

## Abstract

The present study examines the potential impact of a mnemonic acronym on the learning, the execution, the resilience toward interruptions, and the mental representation of an eight-step procedural task with sequential constraints. 65 participants were required to learn a sequential task, including eight different steps which had to be carried out in a predefined sequence. 33 participants were provided with the acronym “WORTKLAU” as a mnemonic to support the learning and execution of the task and the other 32 participants had to learn and execute the task without such support. Each letter of the acronym coded one step of the task, involving a binary decision about a certain property of the complex stimulus. In 60 out of 72 trials of the task, participants were interrupted between different steps, and had to perform a 2-back interruption task for 6 or 30 s, after which they had to resume the procedural task as quickly as possible at the correct step. Learning times, performance in uninterrupted trials, and post-interruption performance measures were analyzed. Results of Experiment 1 suggest that the mnemonic acronym enhanced learning of the task sequence, and provide some evidence for a hierarchical mental representation of the task, resulting in faster resumption times at certain steps of the procedure after an interruption. In Experiment 2 the internal structure of the acronym was even emphasized by a hyphen at the borders of the two words included in the acronym (WORT-KLAU). This improved the resilience toward interruptions at the border step of the procedure significantly. Our results provide evidence for beneficial effects of mnemonic acronym particularly for the learning of a sequential procedural task. In addition, they suggest that the structure of mnemonic acronym directly impacts the mental representation of a task. Finally, they show that mnemonic acronyms could be used to improve the resilience toward detrimental effect of interruptions, at least at certain task steps of a procedural task.

## Introduction

Accomplishing a complex task in everyday life or professional settings often requires to remember how to conduct a procedure that consists of a sequence of steps, which have to be performed in a predefined order. A simple example from everyday life is the sequence of actions needed to make boiled eggs. To get the eggs right, one needs to follow a sequence of steps, and any commission of sequence errors that is deviating from the right order (e.g., putting the eggs in the water before it boils) or omitting to perform a step (e.g., missing to pierce the egg before putting it in the boiling water) may compromise the result. Admittedly, the procedure to cook eggs is fairly easy and consequences of sequence errors in this example are only minor. However, there are other (professional) settings where committing sequence errors while performing a procedural task can have much more serious consequences. Examples are the execution of procedures by pilots on the flight deck, by physicians in an emergency department, or by nurses providing medication in a hospital. Here committing an error may have fatal consequences, which can hardly be corrected, and this risk is even elevated in case of interruptions which have frequently been observed in these settings (Latorella, 1996; Dismukes et al., 1998; Drews, 2007; Scott-Cawiezell et al., 2007; Westbrook et al., 2010). As a countermeasure ensuring the correct execution of procedural tasks and making them more resilient toward interruptions, they often are supported by different sorts of checklists, which shall reduce memory demands and prevent the commission of sequence errors, primarily the missing of important steps (Latorella, 1999; Loukopoulos et al., 2001, 2003). However, checklists are not always available and there are number of instances where even important and safety-critical procedures have to be performed based on memory only (i.e., so called memory items in aviation, Hunt, 1988; Au, 2005). This provides a number of cognitive challenges similar to order memory and serial recall (e.g., Henson, 1998; Hurlstone et al., 2014), including initial learning of the sequence, retaining the sequence across time, and, most important, retrieving the correct order of steps once the procedure has to be executed. According to some authors, the latter is assumed to involve a so-called *placekeeping* process, i.e., monitoring the progress within a procedural task by keeping track of completed and to-be-executed steps (Carlson and Cassenti, 2004; Trafton et al., 2011; Hambrick and Altmann, 2015).

Research from serial learning and recall suggest that these challenges might effectively be supported by the use of mnemonic techniques. It has been shown that the support in organizing the to-be-remembered material in the learning phase enhances learning, has long-term effect on retention of material, and leads to better performance in the recall phase by providing hierarchical organization of the learnt material (e.g., Miller, 1956; Bower, 1970; Bellezza, 1981; Malhotra, 1991; Higbee, 2001). One such technique is the administration of mnemonic acronyms, i.e., pronounceable phrases or words where each letter represents an item that has to be remembered in the order given by the phrase (e.g., first letter mnemonic; Malhotra, 1991; Higbee, 2001). Use of acronyms for memorizing items in a serial order is widely present in education (Cook, 1989; Miller and Mercer, 1993; Stalder, 2005), and in clinical practice (Bortle, 2010). Also, it was found that people voluntary develop acronyms and organize information in chunks (Cook, 1989; Bower, 1970; Blick and Waite, 1971; Blick et al., 1972; Gruneberg, 1973; Bortle, 2010), which also points to the potentially positive effects of such mnemonic for order learning and recall.

Applied to procedural tasks that need to be performed from memory, the provision of acronyms composed of letters which represent the different steps might have at least three beneficial effects. First, it might enhance learning, retention, and retrieval of the steps in correct order. This is suggested by early studies demonstrating advantages of mnemonic acronyms on the learning and reproduction of verbal material (Nelson and Archer, 1972; Stalder, 2005). Positive effects of mnemonic acronyms were shown particularly in situations where the order of items had to be learned and retrieved (Nelson and Archer, 1972; Morris and Cook, 1978), whereas usually no effects were found where the identity of individual items needed to be retrieved (Nelson and Archer, 1972; Morris and Cook, 1978; Carlson et al., 1981). The specific benefit of mnemonic acronyms for memorization of item order, but not item identity, might account for inconsistent findings regarding positive effects of mnemonic acronyms in verbal learning (Boltwood and Blick, 1970; Gruneberg, 1973; Cook, 1989). For that reason, it seems at least plausible that the availability of acronyms would also support learning the correct order of different steps constituting a procedural task.

Second, the availability of an acronym might also increase the execution speed of the different steps of a procedural task, i.e., serve as a process mnemonic tool (Higbee, 1987; Manalo, 2002). This is expected, because the availability of a pronounceable acronym provides a cuing structure whose inherent links between the different letters might strengthen the associations between successive steps (Malhotra, 1991), which in turn could improve the transfer between the steps, leading to an overall increase of speed and accuracy in the execution phase.

Third, it can be assumed that mnemonic acronyms might enhance the resilience of a sequential procedural task toward adverse effects of interruptions. Adverse effects of interruptions, that is, additional time needed to resume a primary task after an interruption (*resumption time*) and elevated risk of committing sequence errors (i.e., skipping or repeating a step), have often been reported when resuming the primary task. Among other factors, the interruption effects depend on the length and complexity of the interruption task (Hodgetts and Jones, 2006; Cades et al., 2007; Monk et al., 2008, see for a review Trafton and Monk, 2007). These effects are often interpreted within the memory for goals model proposed by Altmann and Trafton (2002). The model states that task goals need to be activated in working memory in order to perform a cognitive task. Assuming that cognitive goals underlie the same constraints as other items in working memory, active strengthening is required to reach and maintain sufficient level of activation in order to retrieve the goals successfully. Interruptions of a procedural task cause a decrease of the activation of related task goals, unless the goals are rehearsed while performing the interruption task. Thus, in order to resume the procedural task at the correct position after an interruption, the position within the task needs to be rehearsed during the interruption, and the activation level of the goal related to the correct task step needs to be elevated again, based on internal or external cues. It seems plausible that mnemonic acronyms could provide simple internal cues (e.g., letters instead of words or sentences) for rehearsal and re-activation of task steps, and consequently enhance goal activation in memory. Thus, mnemonic acronyms could be helpful during an interruption, when the goals of the primary task have to be rehearsed in parallel with the execution of the interruption task, as well as after the interruption, when reorienting and re-activation of the primary task goals take place. These effects should be reflected in decreased resumption times and in a decreased risk of sequence errors after interruptions, compared to a situation where no acronym is available.

Given these possible advantages and the available evidence for specific benefits of mnemonic acronyms in terms of order memorization, the provision of mnemonic acronyms to support learning, retention, and retrieval of procedural tasks with sequential constraints seems to be promising. Despite the examples of the use of acronyms to remember and retrieve the correct sequence of steps in a procedure (e.g., decision making procedures, Hörmann, 1994), the performance consequences of this mnemonic technique on learning and execution of procedural tasks were not examined in a systematic manner, thus far, to the best of our knowledge.

A new experimental paradigm, the UNRAVEL paradigm, which principally seems to be suitable to address this question, was recently introduced by Altmann et al. (2014). UNRAVEL is an acronym where each letter represents a step that needs to be executed in response to a complex stimulus, with the letter sequence cueing the correct order of steps of the sequence. The complex task stimuli in this paradigm are composed of a letter, a number, and a box with different features (e.g., font, color, location). The different steps that have to be performed from memory in correct order include responses to a total of seven questions concerning the features of the given stimulus. Thus far, this paradigm has primarily been used to study consequences of interruptions on serial task performance (Altmann et al., 2014, 2017; Altmann and Trafton, 2015). For this purpose, the UNRAVEL task was repeatedly interrupted between steps by a simple interruption task. In order to investigate the performance consequences of these interruptions, the time needed to resume the task (resumption time), the number of sequence errors (i.e., instances where the task was resumed at the incorrect step), and the number of non-sequence errors (i.e., instances where the task was resumed at the correct step, but with the wrong response) were assessed. The obtained results replicated the standard effects in interruption research, namely that the adverse effects of interruptions, i.e., prolonged resumption times and an elevated risk to commit a sequence error, become worse with increasing duration of the interruption task (Altmann et al., 2017). In addition, two aspects of the results suggest that the mnemonic acronym supporting the task, might have made a difference in performing this task. First, even though the UNRAVEL task poses comparatively high memory demands, the observed rates of sequence errors after interruptions were surprisingly low (4–16%), and essentially in the same range or only somewhat higher than the ones usually obtained with much less demanding primary tasks and comparable durations of short interruptions (e.g., Monk et al., 2008). This suggests that the availability of the acronym could have compensated for the higher memory demands of the UNRAVEL task, compared to a condition where the acronym would not have been available. Second, an analysis of performance at different steps of the UNRAVEL task revealed an interesting incidental finding. Namely, the risk of sequence errors was relatively low particularly for the first (U) and last (L) step of the task whereas more sequence errors were committed at the middle steps, even when no interruption preceded the step directly. The authors suggest that the obtained patterns were due to the mnemonic acronym and its structure, which, they assume, have organized the task hierarchically in accordance with the word boundaries of the acronym (Altmann et al., 2014). However, since no control condition (i.e., without an acronym) was included in this interruption research, any conclusions concerning the possible effect of the acronym on interruption performance seem to be hardly conclusive based on the available data of this previous work.

As far as we are aware, there is actually only one UNRAVEL study, thus far, which had included a no-acronym control group. However, this study did not focus directly on the impact of an acronym as mnemonic on performance (Hambrick et al., 2018^1^. Instead, it addressed how individual differences in general ability impacted performance in a placekeeping task with vs. without activation of task-relevant knowledge. Despite the different aims of that study, a look at the data of the different conditions at least suggest that the no-acronym condition was somewhat more demanding than the acronym condition, as participants in the no-acronym group more often consulted the help option than in the acronym group. No differences in overall mean response times (RTs) and rates of sequence error were found between the conditions, though, which is in contrast to the assumption of a generally beneficial mnemonic effect of an acronym on the execution of a serial task. However, because the specific effects of a mnemonic on performance in serial tasks were not the primary aim of this study, the authors just used very general performance measures, not addressing any specific effects of the mnemonic on, for example, learning times, and resilience toward interruptions or task representation. Thus, the conclusions of this study must be considered as very limited with respect to the performance consequences of acronym mnemonics on serial task performance.

The current research aims at a first systematic investigation on the performance effects of a mnemonic acronym vs. no-acronym on learning and performing a procedural task with sequential constraints. For this purpose, we used a German adaptation of the UNRAVEL task and contrasted conditions with and without the mnemonic regarding three different aspects: the time needed for learning the task, the speed and accuracy of executing the task without an interruption, and the potential of the acronym to structure the task and to enhance the resilience of the task (or at least certain steps) toward detrimental performance effects after an interruption. Our adaptation of the UNRAVEL task used a similar task stimulus to the one used by Altmann et al. (2014), but included a total of eight instead of seven task steps, which had to be performed in a certain order. In the acronym condition, the sequence of tasks building the procedure was represented by the acronym WORTKLAU, consisting of two single one-syllable German words, i.e., “Wort” (engl. *word*) and “Klau” (engl. *theft*). Enlarging the procedure to eight steps and using the 2-word acronym was chosen to make the task even more complex and to have an acronym with a salient semantic structure including a central position marked by word boundaries.

In the first experiment, participants performed the primary task either with the support of the acronym (from the learning phase on) or without an acronym. In the latter case, they had to learn the eight steps and their order without any sort of mnemonic technique provided. During performance of the task, we further varied whether or not interruptions of two different lengths occurred at different steps. First, we expected shorter learning times in the acronym condition compared to the condition where no acronym was available. Second, we predicted that having a support of a mnemonic acronym would lead to faster and more accurate execution of the whole sequence of steps compared to the situation without the acronym. This was expected based on the assumption that the sequential associations between steps would be improved by the availability of the acronym. Third, we assumed that availability of the mnemonic acronym would improve the resilience toward interruptions, namely, that resumption times would be faster, and sequence errors at the first step after an interruption would be less frequent, compared to the no-acronym condition. Based on the assumption that acronyms indeed facilitate the rehearsal of where the primary task was interrupted and also provide a salient internal cue to re-activate the task goal at the correct step, this effect should occur independently of the length and position of an interruption. Finally, we assumed that the inherent semantic structure of the acronym would also organize the cognitive representation of the task. That is, we assumed that the mnemonic acronym consisting of two words would facilitate a sort of chunking, i.e., dividing the procedural task into two subunits in accordance with the word boundaries within the acronym. In that case, this should be reflected in a faster learning time and an even higher resilience toward interruption effects, particularly for interruptions occurring at the central position, compared to interruptions elsewhere during the task. This is suggested by the observations of position effects in the UNRAVEL paradigm and previous findings that interruptions are less disruptive if they occur after the completion of subtasks compared to the ones positioned within subtasks (Monk et al., 2002, 2004; Botvinick and Bylsma, 2005; Bailey and Konstan, 2006). Whereas the results of the first experiment allowed for an evaluation of most of these hypotheses, the observed effects were somewhat ambiguous with respect to the effects of the acronym on the mental representation of the task. Thus, a second experiment was run, in which the inherent structure of the acronym was made even more salient by use of a hyphen (“WORT-KLAU”).

## Experiment 1

### Materials and Methods

#### Participants

Seventy four university students, ranging in age from 18 to 30, participated in the study. They were randomly assigned to two groups. 36 participants (23 female, 11 male; *M* = 24.97, *SD* = 2.97) performed the task with support of an acronym and the remaining 38 participants (24 female, 14 male; *M* = 25.16, *SD* = 2.85) performed the task without an acronym. A sample size of 32 participants per group was determined, based on a G-power sample size calculator (Faul et al., 2007) for α = 0.05, power of 0.95, and an effect size of 0.20. Such effect size is in the range of previously reported effect sizes for main performance effects of interruption presence and length (e.g., Altmann et al., 2014, 2017). However, no predictions regarding the sizes of specific effects of providing an acronym could be drawn from previous studies and, thus, were only assumed to be in the same range. Participants were recruited through a web portal of Technische Universität Berlin. For participation in the experiment, a course credit or monetary compensation were offered.

#### Tasks

**FIGURE 1:**
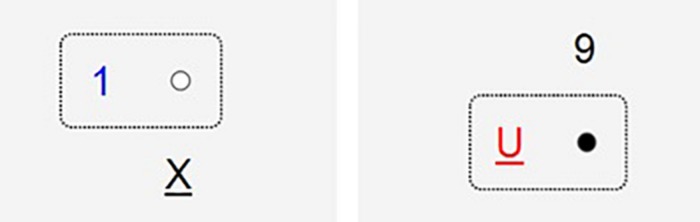
Two examples of task stimuli of the primary WOTRKLAU task.

The primary task was a German adaptation of the UNRAVEL task introduced by Altmann et al. (2014). It follows the same general approach and objectives as the original task, but also takes into account experiences of previous research with this task (Altmann et al., 2014; Altmann and Trafton, 2015). As the UNRAVEL task, the German version also requires participants to respond to a complex stimulus with a number of sequential responses which have to be performed from memory in a predefined order. The stimuli of the primary task correspond to the original stimuli of the UNRAVEL task, with features adapted to a new and enlarged set of choice rules that have to be applied in a given sequence without any cues. That is, each stimulus consists of a dot, a number (1, 2, 3, or 9), a letter (A, B, U, or X) and a box, which differ according to eight different features: color of the dot (white or black), font style of the number (underlined or not), color of the letter/number (red or blue), position of the letter/number outside of the box (above or below), sound of letter (consonant or vowel), font style of the box (dotted or lined), position of the letter in the alphabet (near to the beginning or the end), and parity of the number (odd or even) (see Figure 1).

In response to the stimulus, the participant has to go through a sequential list of eight choice rules, corresponding to the different features, and to type the correct responses in a standard keyboard in a prescribed order. As a mnemonic technique to support learning, retention, and correct execution of the sequence, the WORTKLAU acronym was used. It can be considered as a sort of a first-letter mnemonic representing the sequence of operations of the primary task by the respective first letter of one of the response options, corresponding to the logic of the acronym in the UNRAVEL task. The choice rules, the corresponding responses and the association with the acronym are shown in Table 1.

**TABLE 1:** List of steps, choice rules, and possible answers in WORTKLAU task translated from German to English. Possible answers that form the acronym are provided in both German (direct link to the acronym by first letter of one of the alternatives) and English.

**Step**	**Choice rules**	**Possible responses to be typed in the keyboard**	
1	Dot is white or black	W (*w*eiss/White)	S (schwarz/black)
2	Letter is without or with line	O (*o*hne/without)	M (mit/with)
3	Letter/number is red or blue	R (*r*ot/red)	B (blau/blue)
4	Letter/number is below or above the box	T (*t*iefer/below)	H (hoeher/above)
5	Letter is consonant or vowel	K (*K*onsonant/consonant)	V (Vokal/vowel)
6	Box consists of lines or dots	L (*L*inien/lines)	P (Punkte/dots)
7	Letter is at the beginning or end of alphabet	A (*A*nfang/beginning)	E (Ende/end)
8	Number is odd or even	U (*u*ngerade/odd)	G (gerade/even)

Compared to the UNRAVEL paradigm, the number of steps to be performed in response to each stimulus has been enlarged by one to a total of eight steps in the German WORTKLAU adaptation. This difference has two important consequences: the memory demands of the WORTKLAU task are even higher than in the UNRAVEL task and the acronym is composed of two single words of the same length (i.e., WORT and KLAU; corresponding to the English words *word* and *theft*), which provides a semantic structure to the acronym by dividing it in two parts. The latter makes it possible to study possible effects of the acronym structure on the task execution in a controlled way.

A numerical 2-back task (Moore and Ross, 1963) was used as interruption task. In this task, participants are presented with series of single numbers and need to respond when a presented number equals the one presented two places before. The task places relatively high demands on working memory by requiring a running memory update with each new number presented. It has been used in other interruption research before in order to suppress or at least hinder active rehearsal of where an interruption occurred in a primary task (e.g., Monk et al., 2004, 2008).

#### Procedure

Participants were tested individually in the Human Performance Laboratory of the Chair of Work, Engineering and Organizational Psychology at Technische Universität Berlin. After signing an informed consent and filling in a demographic questionnaire addressing basic biographic characteristics (e.g., age) and relevant experiences (e.g., typing skills), participants were introduced to the WORTKLAU task. In the no-acronym group, the pre-defined order of choice rules was presented, but the sequence of response options was mixed so that forming an acronym from them was not obvious. In contrast, the acronym group was introduced to the mnemonic acronym as support for memorizing the different choice rules in the correct order. Afterward, in both groups followed a short practice phase including five trials of the task, which had to be performed with support of a handout describing the sequence of choices to be made. Immediate feedback on accuracy was provided on the screen after each response. After the practice trials, participants continued with reading of instructions and familiarizing with the interruption task. After the 2-back interruption task was introduced, a short practice trial (1 min) followed.

After this familiarization phase, participants had to pass a knowledge test addressing the procedure and choice rules of the sequential WORTKLAU task. However, before taking this test they could take as much time as they needed to learn the sequence. Participants, who then passed the knowledge test, directly proceeded to the final training without feedback, which consisted of eight WORTKLAU trials with five trials being interrupted after different steps. All other participants got additional learning time before they repeated the knowledge test and could start with the final training block. All participants passed the knowledge test at the second try.

After the final training block, participants had a short break that was followed by the experimental data collection. This main part of the experiment consisted of three experimental blocks, with 24 WORTKLAU trials per block, i.e., 72 trials in total. In each block, 20 trials were interrupted. Interruptions could occur at five different positions in the WORTKLAU sequence (i.e., before steps R, T, K, L, A), each interruption lasting for either 6 or 30 s. That is, each Position × Length combination of interruptions was presented twice per block. The remaining four trials per block, i.e., 12 trials in total, were not interrupted. These were used for assessing effects of the acronym on uninterrupted performance and also used as baseline for calculating interruption effects. Interrupted and uninterrupted trials were mixed randomly. Participants were instructed to proceed as quickly and accurately as possible through the different steps of the WORTKLAU task. In case of errors, they should not correct them, but continue working through the sequence. The interruption task always appeared immediately upon the response to one of the steps of the WORTKLAU task, and replaced the WORTKLAU stimulus fully. During the 2-back interruption task, one number at a time was presented in the center of the screen as a part of short (4 items) or long (20 items) series with a presentation rate of 1.5 s. Immediately after the last item of the 2-back series, the stimulus of the primary task was presented again, and participants were required to resume the primary task as soon as possible at the correct step, i.e., the step that should have followed the last performed step before the interruption. After the last step of a trial in the primary task was performed and before the new stimulus was shown, a blank screen appeared for 300 ms. On average, a complete experimental session lasted 90 min. Before leaving, with each participant a structured interview was conducted, which addressed strategies they used for learning and execution of the task (e.g., “Did you try to divide the task sequence into different parts?,” “Was any position for you especially easy to resume after an interruption?”). In addition, participants subjectively assessed their own performance in the primary and the interruption tasks on simple four-point Likert scales.

#### Design

For examining the effects of a mnemonic acronym on learning times, on overall performance in the uninterrupted trials of the primary task, and on post-interruption performance, we contrasted the performance in the acronym group working with support of the WORTKLAU acronym, with the performance in the no-acronym (control) group.

For investigating the effects of the mnemonic acronym on resilience toward interruptions a 2 (Group) × 2 (Length) × 5 (Position) mixed factorial design was used. The first factor was defined as a between-subjects factor representing the acronym and no-acronym groups. The second factor was defined as a within-subjects factor, representing the length of interruption (6 vs. 30 s). The third factor was again a within-subjects factor and included five levels corresponding to the position in the sequence of response where an interruption occurred (before steps R, T, K, L, A).

#### Dependent Variables

A set of overall eight performance measures were used to assess the impact of the acronym on different aspects of performance including learning, performance during uninterrupted trials, and consequences of interruptions:

##### Learning time

This variable was used to assess a possible impact of the acronym on the time needed to learn the correct sequence of choice rules of the primary task. It was defined as the time passed between the end of the first familiarization phase and the successful pass of the knowledge test, including the extra time needed if the first trial of the knowledge test failed. Operationally it was measured based on the time stamps sampled in the logfile of the experiment, indicating the end of the last 2-back practice trial and the beginning of the final practice block, respectively. In order to be able to control for differences in pure reading speed, the time needed for reading the instructions in the familiarization phase, defined by the time passed between the end of the first practice block of WORTKLAU task and beginning of the 2-back training trials, was also assessed via time stamps sampled in the logfiles of the experiment.

##### Completion time

Completion time was defined as the mean of RTs needed to complete the different steps of the primary WORTKLAU task in trials where no interruptions occurred. For the first step of each trial, RTs were defined as the time (in ms) passed from the occurrence of the new task stimulus until the first response provided. For all following steps, the RT was assessed by the length of inter-response interval (IRI, in ms), elapsed since the preceding response. Only the steps answered correctly were included in this measure.

##### Sequence errors

This measure was defined as the overall mean proportion of all responses to the different steps within uninterrupted trials, where a participant deviated from the prescribed order of the steps, by either missing the steps (e.g., going directly from the W to the R step) or repeating a step.

##### Non-sequence errors

This measure was defined as the overall mean proportion of all responses to the different steps within uninterrupted trials, where a participant provided a response to a given step at the correct position of the trial, but the response was false (e.g., the stimuli presented contained a white dot, but the participant pressed the S instead of W key).

##### Resumption time

Resumption time was defined as the time needed to return to a certain step of the primary task after an interruption. Based on all interrupted trials, it was calculated for each given post-interruption step (R, T, K, L, or A) individually, by subtracting the mean inter-response-interval for this step in the uninterrupted trials from the time passed between the reappearance of the primary task stimulus and the response to this step on the keyboard after an interruption. Only correct responses were considered for this measure.

##### Post-interruption sequence errors

This measure included the proportion of sequence errors (i.e., omitting or repeating a step) occurring at the different steps after an interruption.

##### Post-interruption non-sequence errors

This measure included the proportion of non-sequence errors (i.e., falsely responding to the correct step) occurring at the different steps after an interruption.

##### Interruption task performance

Interruption task performance was measured through correct hits, when participants responded to the stimuli in the task rightfully, and correct rejections, when participants correctly did not respond to the stimuli presented, and it was expressed as a percentage.

In addition to these performance measures a number of further, mainly explorative variables were derived from the structured post-experimental interview, including percentages of different chunking strategies deliberately applied by the participants. Finally, a set of control variables used to identify possible basic differences between the experimental groups included subjective ratings of performance in the primary and in the interruption tasks, and selected items of the demographic questionnaire, like age and subjective ratings of typing proficiency.

### Results

A total of nine participants were excluded from further analyses for either systematic non-sequence errors in the K step (consonant-vowel) of the trial, for using non-mnemonic strategies to conduct the task (i.e., fingers pointing to the correct answer at the keyboard during the interruption), or for inability to analyze all reaction times per situation due to the high number of errors. Thus, the results presented in the following are based on the data of 33 participants in the acronym group and 32 participants in the no-acronym group. The two groups neither differed in age [*M* = 24.97 in the acronym and *M* = 25.38 in the no-acronym group, *t*(63) = 0.55, *p* = 0.58], nor in their proficiency of typing skills [2.73 vs. 2.94, *t*(61.62) = 1.18, *p* = 0.24]. In addition, the two groups also did not differ with respect of their subjective rating of their performance in the primary task [2.94 vs. 3.03, *t*(64) = 0.64, *p* = 0.52] and in the interruption task [2.21 vs. 2.34, *t*(64) = 0.88, *p* = 0.38].

With regard to RT measures in the uninterrupted trials, all RT shorter than 500 ms or larger than 3 standard deviations (SD) from the mean, calculated for each step and each participant, were excluded, resulting in excluding 0.03% RTs in the acronym group, and 0.05% in the no-acronym group. For post-interruption, RTs (between the re-occurrence of the primary task stimulus after interruption and the first response), also all times shorter than 500 ms were excluded, resulting in exclusion of 0.03% in the acronym and 0.04% in the no-acronym group.

#### Learning Time

##### Learning time

All learning and reading times that were 3 SD above or below the group means were excluded from the analysis. This resulted in the suspension of one participant due to a long reading time in the acronym group, and three participants due to long learning and reading times in the no-acronym group. In the acronym group, the mean learning time of the remaining participants was 910.50 s (*SD* = 145.42) compared to 1150.52 s (*SD* = 320.17) in the no-acronym group. An analysis of covariance (ANCOVA) with group as fixed factor and reading times as covariate, revealed a significant difference in learning times between the groups, *F*(1,58) = 13.53, *p* = 0.001, ${\text{η}}_{\text{p}}^{2}$ = 0.19, whereas reading time was not statistically significant, *p* = 0.21.

#### Uninterrupted Primary-Task Performance

A complete overview of the performance measures calculated for each step of the primary task in the uninterrupted condition, together with the three derived overall performance scores on trial level are shown in Table 2.

**TABLE 2:** Means and standard errors (in brackets) of response times, proportion of sequence errors, and proportion of non-sequence errors at each step of the task in uninterrupted trials. In addition, resulting mean completion times and mean overall proportion of sequence and of non-sequence errors are shown for both conditions at the bottom of the table.

	**Acronym group**			**No-acronym group**		
		
**Step**	**Response time**	**Sequence errors**	**Non-sequence errors**	**Response time**	**Sequence errors**	**Non-sequence errors**
W	2361 (166)	0.005 (0.006)	0.005 (0.004)	2405 (169)	0.012 (0.006)	0.011 (0.004)
O	2075 (152)	0.004 (0.002)	0.017 (0.016)	2135 (154)	0.001 (0.002)	0.032 (0.016)
R	1899 (178)	0.008 (0.006)	0.000 (0.000)	1874 (180)	0.011 (0.006)	0.000 (0.000)
T	2284 (187)	0.008 (0.008)	0.008 (0.005)	1987 (190)	0.017 (0.008)	0.009 (0.005)
K	2919 (161)	0.013 (0.006)	0.018 (0.010)	2765 (164)	0.016 (0.006)	0.039 (0.010)
L	2318 (229)	0.018 (0.008)	0.013 (0.006)	2603 (233)	0.038 (0.009)	0.010 (0.006)
A	2651 (196)	0.017 (0.017)	0.010 (0.005)	3117 (199)	0.057 (0.018)	0.006 (0.005)
U	1611 (88)	0.002 (0.012)	0.024 (0.010)	1445 (90)	0.034 (0.012)	0.032 (0.011)
Overall mean	2265	0.009	0.012	2291	0.023	0.017

##### Completion time

Completion times for the different steps in the 12 WORTKLAU trials without interruptions were only descriptively faster in the acronym (*M* = 2265 ms; *SE* = 124) than in the no-acronym group (*M* = 2291 ms; *SE* = 99). This corresponds to an average time needed to complete a whole WORTKLAU of 18.12 and 18.33 s, respectively. A *t*-test contrasting the mean completion times in both conditions did not reveal the difference as significant, *t*(63) = 0.17, *p* = 0.87.

##### Sequence errors

As expected, the mean proportion of sequence errors committed at the different steps of the WORTKLAU task was about half in the acronym group (*M* = 0.009, *SE* = 0.005), compared to the no-acronym group (*M* = 0.023, *SE* = 0.005). However, the proportions of sequence errors were very low in both groups and contrasting these means by a *t*-test the difference just failed to reach the usual level of significance, *t*(38.74) = 1.84, *p* = 0.074.

##### Non-sequence errors

Non-sequence errors followed the same pattern as sequence errors, being lower in the acronym group (*M* = 0.012, *SE* = 0.015) than in the no-acronym group (*M* = 0.017, *SE* = 0.035). However, also this difference was too small to reach statistical significance, *t*(63) = 0.83, *p* = 0.41.

#### Performance in Interrupted Trials

An overview of performance measures calculated for each post-interruption step of the primary task in the conditions with short and long interruptions is shown in Table 3.

**TABLE 3:** Means and standard errors (in brackets) of post-interruption performance measures (response time, resumption time, proportion of sequence, and proportion of non-sequence errors) separately for each position of interruption and both interruption lengths in the acronym and no-acronym group.

		**Acronym group**				**No-acronym group**			
			
**Length**	**Position (Step)**	**Response time**	**Resumption time**	**Sequence error**	**Non-sequence error**	**Response time**	**Resumption time**	**Sequence error**	**Non-sequence error**
Short	2 (R)	4350 (332)	2451 (314)	0.056 (0.024)	0.006 (0.004)	4376 (367)	2364 (318)	0.113 (0.024)	0.000 (0.004)
	3 (T)	3892 (279)	1607 (275)	0.040 (0.017)	0.000 (0.004)	4332 (309)	2361 (279)	0.091 (0.018)	0.005 (0.004)
	4 (K)	4157 (269)	1237 (264)	0.040 (0.019)	0.000 (0.009)	4468 (297)	1637 (268)	0.064 (0.019)	0.034 (0.009)
	5 (L)	4812 (394)	2494 (370)	0.073 (0.025)	0.005 (0.005)	4494 (436)	2128 (376)	0.094 (0.025)	0.005 (0.005)
	6 (A)	4238 (300)	1587 (307)	0.057 (0.018)	0.005 (0.010)	5483 (332)	2123 (312)	0.057 (0.019)	0.011 (0.006)
Long	2 (R)	5294 (479)	3395 (459)	0.278 (0.038)	0.000 (0.000)	6444 (530)	4161 (466)	0.231 (0.038)	0.000 (0.000)
	3 (T)	5714 (499)	3429 (507)	0.162 (0.029)	0.000 (0.005)	6431 (551)	4484 (515)	0.178 (0.030)	0.008 (0.005)
	4 (K)	5425 (444)	2505 (440)	0.207 (0.033)	0.030 (0.018)	6626 (491)	3596 (447)	0.131 (0.034)	0.026 (0.019)
	5 (L)	6709 (468)	4391 (506)	0.291 (0.046)	0.005 (0.004)	6122 (517)	3541 (514)	0.219 (0.047)	0.000 (0.004)
	6 (A)	6436 (560)	3785 (535)	0.244 (0.042)	0.005 (0.010)	6825 (619)	3221 (543)	0.290 (0.043)	0.018 (0.010)

##### Resumption times

**FIGURE 2:**
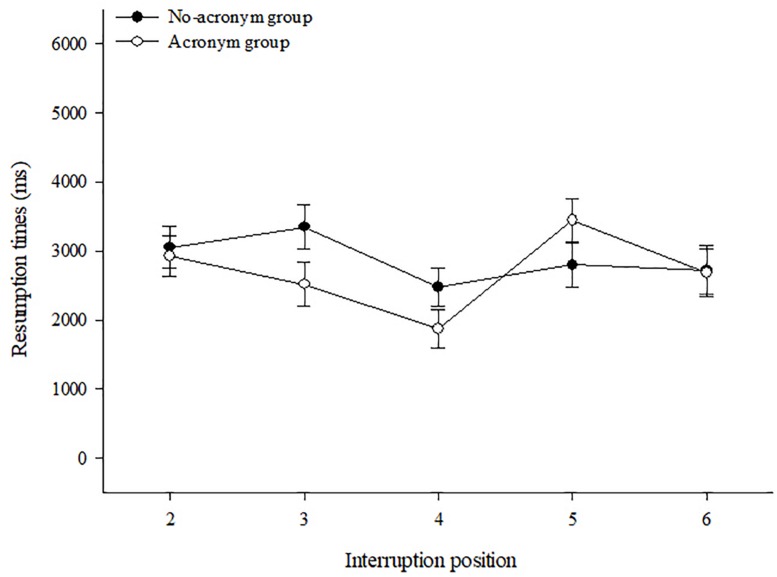
Resumption times and standard errors of the acronym and no-acronym group for different interruption positions.

The 2 (Group) × 2 (Length) × 5 (Position) ANOVA, revealed significant main effects of length of interruption *F*(1,63) = 105.77, *p* < 0.001, ${\text{η}}_{\text{p}}^{2}$ = 0.63, and position of interruption, *F*(4,252) = 4.52, *p* = 0.002, ${\text{η}}_{\text{p}}^{2}$ = 0.07, as well as a Group × Position interaction, *F*(4,61) = 3.05, *p* < 0.018, ${\text{η}}_{\text{p}}^{2}$ = 0.05. No other effects became significant, all *p* > 0.10. As expected, long interruptions led to longer resumption times (*M* = 3651 ms, *SE* = 256) compared to short ones (*M* = 1999 ms, *SE* = 145). The effects of group and interruption position on the resumption time, including their interaction, are shown in Figure 2. As becomes evident, resumption times were different, dependent on the position at which the interruptions occurred, with the shortest resumption times in both groups when the interruption occurred at the center position. However, this latter effect was somewhat more pronounced in the acronym group than in the no-acronym group. A planned *t*-test for paired samples contrasting the resumption time for interruptions at the central position (*M* = 1765 ms, *SE* = 277) and the mean of all other positions (*M* = 2710 ms, *SE* = 265) revealed a significant effect in the acronym group, *t*(32) = 3.24, *p* = 0.003, whereas the same comparison failed to become significant in the no-acronym group (*M* = 2591 ms, *SE* = 325 vs. *M* = 2948, *SE* = 296), *t*(31) = 1.41, *p* = 0.17. However, also the marked increase of resumption times for interruptions at position #5 (“L” step) compared to the center position, which is visible in the acronym group, but absent in the no-acronym group, might have contributed to the interaction effect.

##### Post-interruption sequence errors

**FIGURE 3:**
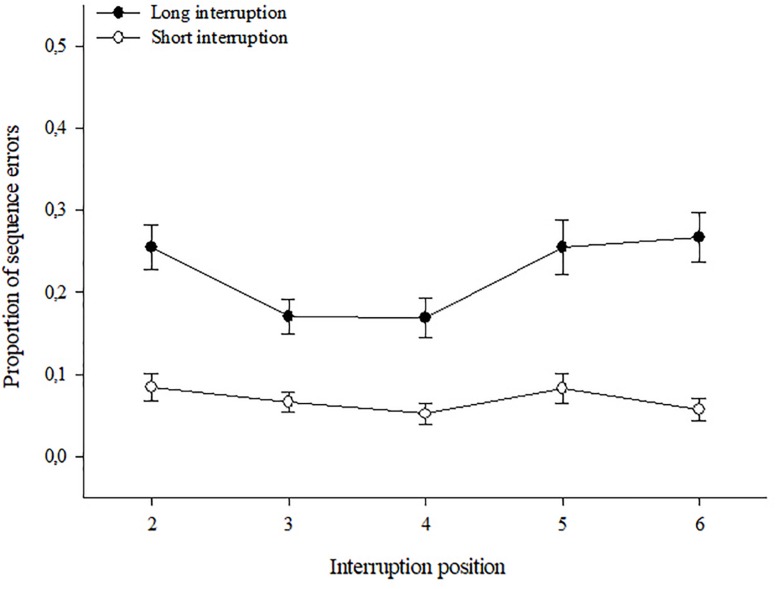
Proportion of sequence errors and standard errors of the acronym and no-acronym groups together at different interruption positions.

The 2 (Group) × 2 (Length) × 5 (Position) ANOVA revealed significant main effects of interruption length, *F*(1,63) = 110.73, *p* < 0.001, ${\text{η}}_{\text{p}}^{2}$ = 0.64, and of position *F*(4,252) = 3.72, *p* = 0.006, ${\text{η}}_{\text{p}}^{2}$ = 0.06, as well as a significant Length × Position interaction, *F*(4,252) = 2.97, *p* = 0.02, ${\text{η}}_{\text{p}}^{2}$ = 0.04. No other effects became significant, all *p* > 0.06. In accordance with the result obtained for resumption times, long interruptions led to higher rates of sequence errors (*M* = 0.223, *SE* = 0.017) compared to short ones (*M* = 0.068, *SE* = 0.007), and this effect emerged independently of the acronym availability. The mean rates of sequence errors reflecting the Length × Position interaction are shown in Figure 3. As becomes evident, in case of short interruptions, the rate of post-interruption sequence errors was generally low and did not vary much dependent on the position of the interruption. However, for long interruptions, the rate of sequence errors differed across positions, with the lowest error rates after interruptions at the positions #3 and #4 (center). A *post hoc t*-test for paired samples contrasting the mean error rate at the central position with the mean of all other positions was conducted for the two interruption lengths separately. With short interruptions, no differences were found, *t*(64) = 1.34, *p* = 0.18, whereas the analysis for long interruptions revealed less sequence errors at the central position (*M* = 0.170, *SE* = 0.024) compared to the mean of all others (*M* = 0.240, *SE* = 0.018), *t*(64) = 2.72, *p* = 0.008. However, whether or not an acronym was available to support the execution of the procedure did not make a difference.

Previous studies have shown that interruptions may not only raise the risk of sequence errors, but specifically sequence errors in form of repeating a step (in the terms used by Altmann et al., 2014 perseveration error) instead of skipping a step (anticipation error), whereas the latter was found to be more characteristic in uninterrupted trials. Thus, we performed an exploratory *post hoc* analysis investigating whether the provision of a mnemonic acronym would make a difference in this respect. A 2 (Group) × 2 (Context: with vs. without interruption) × 2 (Error type: repeating vs. skipping) ANOVA only revealed a significant main effect of context, *F*(1,63) = 135.51, *p* < 0.001, ${\text{η}}_{\text{p}}^{2}$ = 0.68, and a Context × Error type interaction, *F*(1,64) = 5.74, *p* = 0.02, ${\text{η}}_{\text{p}}^{2}$ = 0.08. Overall, participants more often committed a sequence error after an interruption (*M* = 0.073, *SE* = 0.005) than in the uninterrupted trials (*M* = 0.010, *SE* = 0.002). In context of uninterrupted trials, participants were more likely to skip (*M* = 0.016, *SE* = 0.003) than to repeat a step (*M* = 0.005, *SE* = 0.001), while an opposite tendency was found in the post-interruption context (*M* = 0.068, *SE* = 0.006 vs. *M* = 0.079, *SE* = 0.008). Neither the main effect of group, nor any interaction effect of group and the other factors became significant (all *p* > 0.35).

##### Post-interruption non-sequence errors

The mean rate of post-interruption non-sequence errors was generally low (<0.03) in both groups with only few variations induced by the experimental conditions. Thus, we assumed the variations reflected in this measure as just random and resigned to analyze non-sequence errors statistically.

##### Interruption task performance

Mean accuracy in the interruption task was ranging between 82 and 100% (*M* = 93.95%, *SE* = 0.77) in the acronym group, and between 68 and 99% (*M* = 91.78%, *SE* = 1.02) in the no-acronym group. Despite the trend of somewhat lower performance in the no-acronym group, a *t*-test for independent samples showed no significant differences in 2-back task between the groups, *t*(64) = 1.71, *p* = 0.09. In order to examine possible relationships between the performance in the interruption task and the post-interruption performance, a Pearson’s product-moment correlation coefficient was computed for each group separately. In the acronym group, the accuracy in the interruption task did not correlate with the mean resumption time, *r* = 0.10, *p* = 0.60, *n* = 33, nor with the mean proportion of post-interruption sequence errors, *r* = 0.03, *p* = 0.86, *n* = 33. However, in the no-acronym group, a significant correlation between the accuracy in the interruption task and the mean resumption time was found, *r* = 0.62, *p* < 0.001, *n* = 32. That is, higher accuracy in the interruption task was related to longer resumption times after the interruption. No correlation between interruption task performance and mean proportion of post-interruption sequence errors was found, *r* = −0.04, *p* = 0.81, *n* = 32.

#### Post-experimental Interview

##### Use of chunking strategy

Chunking the task into subtasks in the learning and the execution phase was the common strategy in the acronym group, employed by 79% participants: 33% of participants reported to have split the task into two halves corresponding to the two words building the acronym (WORT – KLAU) and 39% of the sample split the acronym in three parts (WO – RT – KLAU), also based on the semantic structure of the acronym, but including the word “wo” (engl. “where”) as a separate part. The remaining 6% reported to have used some other sort of chunking strategy (e.g., 4 × 2 steps). On the other hand, only 34% of participants in the no-acronym group employed task chunking as a strategy: 13% of participants split the task in two halves, 10% of participants split the task in 4 × 2 steps, and the rest (9%) used some other way of chunking the task. On a descriptive level, shorter learning times emerged in a subgroup of participants who employed some kind of task chunking compared to a subgroup who did not, within each experimental group (acronym group: 899.73 vs. 990.00 s; no-acronym group: 988.44 vs. 1241.45 s).

##### Ease of resumption

Participants also reported whether any interruption position was particularly easy to resume. In the acronym group, 30% of participants reported the central position as especially easy to resume, and 36% of participants reported the central position in addition to some other as being particularly easy. In contrast, only 22 and 13% participants of the no-acronym group reported the same benefit of these positions. The remaining 22 and 19% of participants in the acronym and in the no-acronym group, respectively, did not reported any position as specifically easy to resume, and the rest of participants reported some other positions or their combinations.

### Discussion

The aims of the present research were to investigate effects of the availability of a mnemonic acronym on learning and execution of a procedural task, the resilience toward detrimental effects of interruptions, and the impact of the structure of a mnemonic acronym on the mental representation of the task.

Let us first consider the effects of learning and execution of the eight-step procedure in the uninterrupted trials. Based on knowledge gained from research on memory for order (Nelson and Archer, 1972; Morris and Cook, 1978), beneficial effects of the mnemonic acronym were expected to emerge in the time needed to learn the procedure. In accordance with this hypothesis, the acronym group acquired the procedure significantly faster than the no-acronym group. This finding is in line with previous research on memory for order, showing positive effects of mnemonic acronyms on memorization of the order of verbal items (Morris and Cook, 1978). However, no beneficial effects of the acronym were found with respect to completion time and error rates, confirming recent observations in the study of Hambrick et al. (2018). Thus, our additional hypothesis that mnemonic acronyms might also serve as process mnemonics that promote speed and accuracy of task execution through strengthening associations between the steps of the procedure (Malhotra, 1991) was not supported by the data. Obviously, once the procedure was learnt, no additional benefit of the cuing structure was provided by the mnemonic acronym during the actual execution. This suggests that mnemonic acronyms can serve well as learning mnemonics supporting the establishment of declarative knowledge concerning the set and sequence of rules of a sequential task. However, the transfer of this knowledge to the actual active execution of the task (Kieras and Bovair, 1986) does not seem to be supported further by the availability of a mnemonic acronym.

A second aim of the experiment addressed possible effects of mnemonic acronyms on the resilience of executing procedures toward interruptions. More specifically, we assumed that acronyms would generally improve the rehearsal of where in the sequence the interruption occurred and provide cues for a better re-activation of the correct step to-be-performed next after the interruption. The results did not support this assumption, as no differences between the groups were found in terms of overall resumption times and post-interruption sequence errors. This was also reflected in the subjective ratings of primary task and interruption task performance where no significant differences emerged between the two groups. However, the finding of the explorative analysis regarding the different relationship between the accuracy in the interruption task and resumption time in the two groups revealed a more subtle effect of the mnemonic acronym, which suggests that the availability of a mnemonic might have facilitated the rehearsal of primary task goals during the interruption phase. Nonetheless, in the no-acronym group participants who were more accurate in the interruption task needed more time to resume the primary task after the interruption and vice versa, and no such mutual dependence was found in the acronym group. This suggests that the mnemonic acronym could have provided simple rehearsal cues, which helped to reduce possible interference effects between performing the 2-back task and rehearsal of the primary task goal during the interruption phase, i.e., allowed for similar resumption performance independent of how much priority was given to the performance in the 2-back task.

Finally, we expected that the structure of the acronym would affect the mental representation of the task. More specifically, we assumed that the word boundaries included in the acronym consisting of two words (WORT, KLAU) would lead to a chunking of the procedure in at least two parts, based on the meaning of the words within the acronym. This then should be reflected in faster and more accurate post-interruption performance at the central step of the procedure compared to the others. No such effects were expected in the no-acronym group. The results do not seem to be fully conclusive in this regard. Based on the post-experiment interview, the vast majority participants (about 80%) in the acronym group deliberately used the semantic structure of the acronym in one way or the other to divide the procedure in different chunks, whereas only a minority of participants in the no-acronym group reported to do so. Albeit this is in general accordance with our hypothesis, it was not as clearly reflected in the performance data. Here, a significant Group × Position effect emerged for resumption times, but was not easy to explain since also marked differences at positions other than the central position might have contributed to this effect. Furthermore, no comparable effect was found for the post-interruption sequence errors. Thus, before accepting the hypothesis that providing mnemonic acronyms as a tool to structure the mental representation of tasks, we conducted a second experiment where we made the word boundary within the acronym WORTKLAU even more salient.

## Experiment 2

The results of the first experiment provided some evidence that the semantic structure of the mnemonic acronym affected the mental representation of the task. In order to investigate this possible effect further, we even emphasized the word boundary of the acronym WOTKLAU by introducing a hyphen between the words WORT and KLAU of the acronym in the learning. We expected that such subtle, but salient manipulation would be more effective than just the internal semantic structure of the acronym to structure the mental representation of the task in two halves, corresponding to the word boundary in the central position of the acronym. During the execution of the task, this should be reflected in a considerable higher resilience toward interruptions, specifically reflected in clearly reduced resumption times and sequence error rates for interruptions taking place at the central position (i.e., before step “K”) than all other positions. However, learning times and performance at uninterrupted trials of the primary task are expected to remain unaffected and to replicate the results obtained in Experiment 1 for the acronym group.

### Materials and Methods

#### Participants

Twenty university students (seven female; *M* = 25.65, *SD* = 3.31), ranging in age from 19 to 30, participated in the study. Participants were recruited through a web portal of Technische Universität Berlin. For participation in the experiment, a course credit or monetary compensation were offered.

#### Task and Procedure

Tasks and procedure were the same as in the acronym group in Experiment 1. The only difference regarded the learning phase of the experiment, where the acronym “WORTKLAU” was replaced with “WORT-KLAU.”

#### Design

For investigating effects of the mnemonic acronym with salient central position on resilience toward interruptions a 2 (Length) × 5 (Position) within-subjects factorial design was used. The first factor was defined as a within-subjects factor, representing the duration of interruption (6 vs. 30 s). The second factor was another within-subjects factor and included five levels corresponding to the position in the sequence of response where an interruption occurred.

#### Dependent Variables

Learning times, uninterrupted primary-task performance measures (completion time, sequence, and non-sequence errors), and post-interruption performance measures (resumption times, post-interruption sequence and non-sequence errors, and interruption task performance) were calculated in the same way as in Experiment 1. In addition, the same explorative and control variables as in the first experiment were included, i.e., percentages of different chunking strategies deliberately applied, subjective ratings of performance in the primary and in the interruption tasks, age, and subjective ratings of typing proficiency.

### Results

Due to a high number of errors, leading to missing resumption times in certain conditions, one participant was excluded from the further analyses. The mean subjective ratings of typing speed, primary task performance and interruption task performance of the remaining participants were 2.74 (*SE* = 0.13), 3.0 (.13) and 2.21 (.14), and, thus, replicated the mean ratings of the acronym group of the first experiment almost exactly (2.73; 2.94; 2.21, respectively). Applying the same criteria for outlier correction as in Experiment 1, in total 0.02% single values were excluded from RTs in uninterrupted trials, and 0.02% values from the post-interruption RTs.

#### Learning Time and Uninterrupted Primary-Task Performance

##### Learning time, completion time, sequence, and non-sequence errors

Because no experimental manipulation in this experiment addressed learning time and the primary task performance in uninterrupted trials, only descriptive statistics are reported. Mean learning time was 999.21 s (*SE* = 46.28), which was close to the mean learning time needed in the acronym group of Experiment 1 (910.50 s). Similarly, also the baseline performance scores achieved in the primary task in uninterrupted trials more or less replicated those of Experiment 1. The mean completion time per step in uninterrupted trials was 2716 ms (*SE* = 215) corresponding to the average time needed to complete a whole WORT-KLAU trial of 21.73 s (*SE* = 1.72). The mean errors rates were 0.014 (*SE* = 0.004) for sequence errors and 0.011 (*SE* = 0.002) for non-sequence errors.

#### Performance in Interrupted Trials

##### Resumption times

**FIGURE 4:**
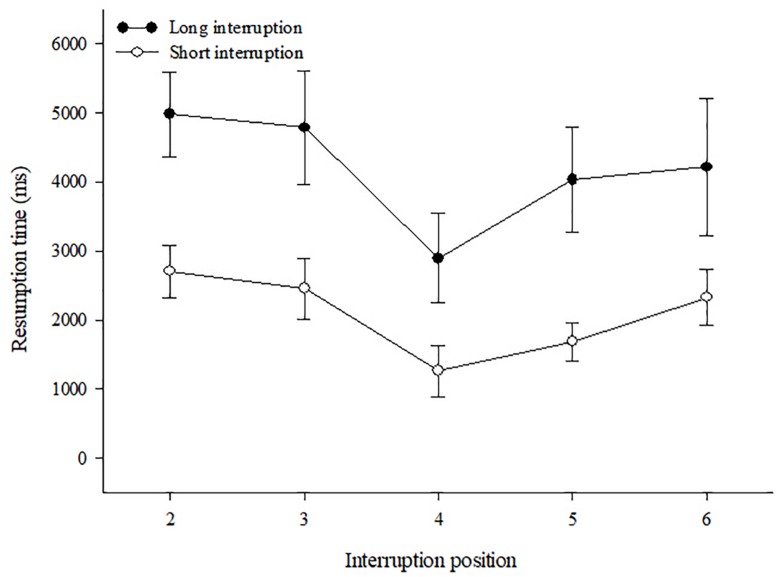
Resumption times and standard errors for short and long interruption length at different interruption positions.

The effects of interruptions on mean resumption times are shown in Figure 4. A 2 (Length) × 5 (Position) ANOVA for repeated measures only revealed the two main effects as significant, Length: *F*(1,18) = 32.27, *p* < 0.001, ${\text{η}}_{\text{p}}^{2}$ = 0.64; and Position: *F*(4,72) = 3.27, *p* = 0.016, ${\text{η}}_{\text{p}}^{2}$ = 0.15. As expected and as becomes evident from Figure 4, long interruptions led to considerably longer resumption times (*M* = 4185 ms, *SE* = 472) than short ones (*M* = 2089 ms, *SE* = 250). In addition, resumption times differed across positions with quickest resumptions after interruptions at the central position. A *t*-test for paired samples contrasting the mean resumption time at the central position (*M* = 2041 ms, *SE* = 448) with the mean of all other positions (*M* = 3222 ms, *SE* = 343), revealed this difference as significant, *t*(18) = 3.21, *p* = 0.005.

##### Post-interruption sequence errors

**FIGURE 5:**
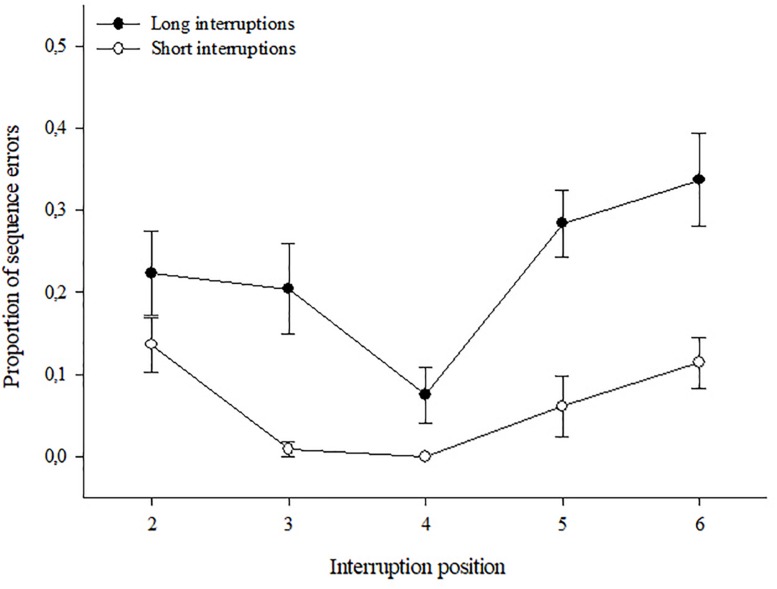
Proportion of sequence errors and standard errors for short and long interruption length at different interruption positions.

Effects of Length and Position of interruptions on post-interruption sequence errors are shown in Figure 5. As becomes evident, again both factors obviously affected the risk to commit such errors, yet in a somewhat different way across positions, dependent on the length of interruption. The ANOVA revealed significant main effects of Length *F*(1,18) = 42.12, *p* < 0.001, ${\text{η}}_{\text{p}}^{2}$ = 0.70, and Position *F*(4,72) = 7.67, *p* < 0.001, ${\text{η}}_{\text{p}}^{2}$ = 0.30, as well as an interaction effect *F*(4,15) = 2.80, *p* = 0.032, ${\text{η}}_{\text{p}}^{2}$ = 0.13. As expected, long interruptions lead to more sequence errors (*M* = 0.225, *SE* = 0.031) compared to short ones (*M* = 0.064, *SE* = 0.014). Regarding the Position effect, it becomes evident from Figure 5 that, independent of the length of interruption the risk of sequence errors was lowest at the central positions with actually perfect performance after short interruptions. This was confirmed by a *post hoc t*-test for paired samples, contrasting mean sequence errors after interruptions at the central position with the mean errors after interruptions at other positions which became significant for both interruption lengths: short interruptions, *t*(18) = 4.33, *p* < 0.001, and long interruptions, *t*(18) = 3.93, *p* = 0.001. However, for short interruptions also the risk of sequence errors after interruptions at the second position was almost zero (*M* = 0.009, *SE* = 0.009), and also this mean differed significantly from the means of positions #2, and #6, all *p* < 0.003.

##### Post-interruption non-sequence errors

Mean error rates of post-interruption non-sequence errors were generally low (<0.06%) and were not further analyzed statistically.

##### Interruption task performance

Mean accuracy in the interruption task was ranging between 82 and 99% (*M* = 92.38%, *SE* = 1.05). Corresponding to the results of the acronym group in Experiment 1, the accuracy in the interruption task did neither correlate with the mean of resumption times, *r* = 0.22, *p* = 0.37, *n* = 19, nor with the mean proportion of post-interruption sequence errors, *r* = −0.18, *p* = 0.46, *n* = 19.

#### Post-experimental Interview

##### Use of chunking strategy

Chunking the task into halves as a deliberately applied strategy during the learning and the execution phase, directly corresponding to the emphasized semantic structure of the primary task, was explicitly reported by 42% of the participants. Only one participant reported some other chunking pattern (3+5). However, 53% of the participants did not report a use of any chunking strategy. In a *post hoc* analysis, *t*-test for independent samples revealed no differences in learning times between the subgroup of participants who used some kind of chunking strategy (1165.11 s) and participants who did not chunk the task (1266.80 s). However, the trend observed was in accordance with the finding in Experiment 1, where the subgroup who employed chunking was faster in learning than the subgroup who did not.

##### Ease of resumption

Thirty nine % of the participants reported the central position as especially easy to resume after an interruption, and 33% of participants reported the central position in addition to some other. In contrast, only 28% of participants reported that no position was particularly easy to resume.

### Discussion

The main objective of Experiment 2 was to examine the effects of a more salient semantic structuring of the mnemonic acronym on the mental representation of the sequential WORTKLAU task. Compared to the acronym used in the first experiment, the two-word structure of the acronym was made more salient by simply including a hyphen at the boundary between the two words WORT and KLAU. It was expected that this would lead to a structured mental representation of the WORTKLAU task, consisting of two parts, represented by the word. This, in turn, was expected to make the task more resilient toward interruptions at the central position, reflected in shorter resumption times and less sequence errors when resuming the primary task after interruptions at the central position, compared to interruption at other steps. The obtained results provide support for this hypothesis. Independent of the length of interruptions, and more clearly visible than in the first experiment, the primary task was resumed faster and more accurately after the interruptions at the central compared to the mean of all other positions. This effect was most marked for long interruptions, where the mean rate of post-interruption sequence errors at the central position dropped to only 8%. For short interruptions, the rates of post-interruption sequence errors were relatively low anyway, but actually zero for all participants at the central position. Taken together, these results confirm the findings obtained in the acronym group of Experiment 1, which already suggested that the semantic structure of an acronym provided as a mnemonic for a sequential task might also affect the structure of mental representation of the task. The fact that this effect was more strongly reflected in the performance measures than in the subjective reports of the participants concerning the use of a deliberately chosen strategy suggest that it can occur without becoming subjectively aware.

## General Discussion

The aim of the present study was to examine the potential of a mnemonic acronym to serve as a learning mnemonic for a sequential procedural task, and to serve as a process mnemonic during the task execution. Moreover, the goal was to investigate the potential of a mnemonic acronym to improve overall resilience toward interruptions by providing an easily accessible cue for rehearsal, as well as to improve resilience toward interruptions at certain steps by providing a structure to the mental representation on the procedure. To our knowledge, this is the first study that has addressed these questions directly in a systematic way.

The results of the two experiments provide direct empirical evidence for the beneficial effects of a mnemonic acronym as a support tool for learning. The two groups provided with different versions of the WORTKLAU acronym in the learning phase needed approximately 5 min less (on average) to learn the rules of the sequential procedure, compared to the participants of Experiment 1 who learned the sequence without the help of an acronym. These effects are in line with early studies on mnemonic acronyms that showed their positive effects in learning and reproduction of verbal material (e.g., Higbee, 2001; Stalder, 2005), especially when the order of items needs to be memorized (Nelson and Archer, 1972; Morris and Cook, 1978), which was the key property of the task in our experiment. The effects suggest that the knowledge gained from serial verbal learning can be transferred directly to the learning of sequential procedural tasks, involving different steps to be performed in prescribed order. However, at least partially, the positive effects may also be explained by the structure of the complex WORTKLAU acronym, which could have enhanced the chunking of the task during learning and execution. Within each experimental group in both experiments, the subgroup that chunked the task was somewhat faster in learning compared to the subgroup that did not report such strategy. Although this difference only emerged descriptively and should not be overemphasized, it at least suggests that learning times in the acronym group did not only benefit from the support of the acronym as cue for coding the order and content of choice rules, but also from its structure supporting a hierarchical task organization. In addition, it cannot be excluded that also indirect benefits of the mnemonic acronyms, e.g., increase motivation to work on the task (Stalder, 2005), might have contributed to the faster learning times in the acronym group.

A more advanced assumption involved the hypothesis that the mnemonic acronym might also serve as a process mnemonic improving the speed and accuracy of a procedural task execution. That is, we expected that the mnemonic acronym would provide a cuing structure, which strengthened the associations between successive steps of the task (Malhotra, 1991). In that case, a faster and more accurate execution of the task sequence would be enabled. This expectation was mainly based on observational and field studies reporting the benefits of mnemonic acronyms for supporting learning, teaching, and executing of procedures (Cook, 1989; Stalder, 2005; Bortle, 2010). However, the data of the two experiments do not support this assumption. In neither experiment, the groups performing the primary task with support of the acronym did outperform the control group of Experiment 1 when performing the task in uninterrupted trials, i.e., both groups achieved the same levels of speed and accuracy. This suggests that the mnemonic acronym did only support the establishment of declarative knowledge in long-term memory, but failed to further support the transfer of the memorized sequence of rules in the sequence of response selections and actions required for the actual execution (Kieras and Bovair, 1986). Theoretically, the execution of such sequential, procedural task is proposed to rely on mechanisms involved in order memory and serial recall (single mechanism theories, e.g., Burgess and Hitch, 1999; Brown et al., 2000; Botvinick and Plaut, 2006) or on a specific placekeeping ability involving two mechanisms – episodic and semantic memory (Trafton et al., 2011; Hambrick and Altmann, 2015). Both groups of theories propose chain associations between the steps, where the execution of one step serves as a prime for the activation and execution of following steps. The results of the present study suggest that, once a sequential procedural task is learnt, with or without the support of an acronym, the sequential associations between successive steps are already strong enough to serve this assumed cueing and priming mechanisms sufficiently, rendering all additional effects of an acronym negligible.

A third set of assumptions concerned the possible effects of a mnemonic acronym to improve the resilience of a procedural task toward interruptions and to affect the structure of mental representation of the task. A general higher resilience toward interruptions was expected based on the assumption that rehearsal of a pending goal during the interruption task would be enhanced by a simple internal cue provided by the acronym, leading to elevated activation of the primary task goal during the interruption (Altmann and Trafton, 2002). In addition, we assumed that the acronym might provide effective cues for reorienting and re-activation the correct step of the primary task after an interruption. However, no differences in post-interruption measures, neither resumption times nor sequence errors, were found between the acronym and the no-acronym group of Experiment 1. This suggests that a mnemonic acronym does not contribute to a better prevention of goal decay during the interruption phase, nor does it seem to be especially helpful as a cue for reactivating the primary task goal after the interruption. However, the observation that the performance in the interruption task and the time needed to resume the primary task were positively correlated across participants in the no-acronym (Experiment 1), but not across participants of the two acronym groups (Experiments 1, 2) suggests that the mnemonic acronym nevertheless affected the rehearsal processes in the interruption phase in some way. More specifically, it enabled participants to better uncouple the processes involved in the 2-back task from rehearsing the relevant primary task goals. Why such effect would not lead to better resumption performance is difficult to explain, though, and the interpretation should be considered with some caution, given that the correlations were based on relatively small number of participants in the different groups and a restricted variance of interruption task performance especially in the two acronym groups.

Even more specific effects on the resilience toward interruptions were expected due to the potential impact of the mnemonic acronym on the organization of the mental representation of a procedural task established during learning. Specifically, it was assumed that the semantic structure of an acronym would guide a sort of hierarchical mental task representation, which in turn would make a procedural task more resilient toward interruption at task steps representing a boundary in the semantic structure of the acronym. This assumption was supported by the data of both experiments. In the first experiment, post-interruption performance in terms of resumption times was better when the interruption was placed between the two separate words building the acronym, compared to interruptions at other steps. When the boundary between the acronym words were made even more salient (Experiment 2), the effect was replicated in both resumption times and post-interruption sequence errors. This finding is in line with previous studies, which examined the relationship between the hierarchical structure of a task and interruption effects (Monk et al., 2002, 2004; Botvinick and Bylsma, 2005; Bailey and Konstan, 2006). They usually found interruptions being less detrimental for performance, if they occurred between subtasks compared to within subtasks. These results were explained by reduced mental workload at subtask boundaries, resulting from previous subtask completion and not yet fully processing the incoming one (Miyata and Norman, 1986; Wickens, 2002). Based on our research, it appears that mnemonic acronyms can induce a hierarchical mental organization of a complex procedural task with sequential constraints in different subtasks even if the task *per se* does not have such structure. However, considering the effect of this hierarchical organization on the resilience toward interruptions one should keep in mind that these effects were considerably smaller compared to the impact of interruption length. Whereas the observed sizes of the effects of position of interruptions ranged between 0.06 and 0.3 across the two experiments, the different lengths of interruptions produced considerable larger effects (0.63–0.70), which could not be completely attenuated by help of the mnemonic in either of the two experiments.

The interruptions in our experiments primarily were used to specifically assess the possible effects of mnemonic acronyms on the resilience of a procedural task toward interruptions. Apart from this, our results also contribute to interruption research in general. Independent of whether or not the mnemonic acronym was available, most of the performance consequences of interruptions previously described from research with the UNRAVEL tasks (Altmann et al., 2014, 2017) were confirmed again in our experiments. That is, resumption times and proportion of post-interruption sequence errors increased depending on the length of interruptions, with mean rates of sequence errors after short and long interruptions closely resembling the ones reported by Altmann et al. (2014, 2017). In addition, the somewhat higher prevalence of erroneous repetition of steps (perseveration errors) versus skipping of steps (anticipation errors) after interrupted compared to uninterrupted steps, previously reported by Altmann et al. (2014) is replicated in our research. This provides converging evidence for these phenomena to cross-validate the previous findings obtained in the UNRAVEL task using our modified German adaptation combined with a different interruption task.

Altogether, to our knowledge, the current study is one of the first attempts to examine extensively effects of mnemonic acronym on learning and execution of procedural task with sequential constraints, as well as resilience toward interruptions in an experimental setting. The results provide support for implementing mnemonic acronyms in the learning phase of a procedural task, as they can promote faster learning. However, once the task is learnt, no additional benefit of the acronym on plain execution of the task would be expected. Furthermore, it seems that a mnemonic acronym can also affect the mental representation of a serial task by dividing it in subtasks, which in turn may lead to a higher resilience toward interruptions at subtask borders. Thus, overall, the results provide evidence of limited and specific advantages of mnemonic acronyms in context of procedural tasks, which should be further consolidated in future research. Moreover, the finding that a hierarchically organized mental representation of a procedural task can help to make this task more resilient toward interruptions at certain positions also raises the question about other ways to achieve such organization. Besides providing mnemonic acronyms, for example, also a segmented learning of a procedure by organizing the steps in pairs or subgroups, or a temporal grouping similar to the one applied in previous research on memory for serial order (e.g., Parmentier and Maybery, 2008) might provide options to yield a hierarchical representation of a task and might be considered in future research.

Limitations of the current study involve the typical limitations of laboratory studies. Our participants were university students, who might be considered to represent an already highly selected population with respect to the level of their cognitive capabilities. However, in the context of the current study this might have made it rather more difficult to find beneficial effects of a mnemonic acronym. In addition, the WORTKLAU task used in our research to simulate a procedural task with sequential constraints certainly is an abstract laboratory task. We just assume that the cognitive demands of this task closely resemble the ones needed in many procedural tasks in everyday environments and applied settings. Nevertheless, the consequences of committing errors in task execution were not quite comparable to typical tasks outside the laboratory. Thus, further research should show whether the effects found in this research can be replicated with more representative samples and more realistic tasks in relevant field settings.

## Data Availability Statement

The datasets generated for this study are available on request to the corresponding author.

## Ethics Statement

The studies involving human participants were reviewed and approved by the Ethics Committee of the Institute for Psychology and Ergonomics, Technische Universtät Berlin [Die Ethik-Kommission des Instituts für Psychologie und Arbeitswissenschaft (IPA) der TU Berlin]. The patients/participants provided their written informed consent to participate in this study.

## Author Contributions

TR and DM conceived the idea, planned the experiments, and contributed to the analysis of the results and writing of the manuscript.

## Conflict of Interest

The authors declare that the research was conducted in the absence of any commercial or financial relationships that could be construed as a potential conflict of interest.

## Footnotes

1 We are grateful to one of the reviewers to make us aware of this study.
